# A metasurface-based electronically steerable compact antenna system with reconfigurable artificial magnetic conductor reflector elements

**DOI:** 10.1016/j.isci.2022.105549

**Published:** 2022-11-12

**Authors:** Vikrant Singh, Mohsen Khalily, Rahim Tafazolli

**Affiliations:** 1 5G Innovation Centre & 6G Innovation Centre (5GIC & 6GIC), Institute for Communication Systems (ICS), University of Surrey, Guildford, UK

**Keywords:** Physics, photonics, engineering

## Abstract

Beyond 5G networks would require newer technologies to deliver a smarter network. In accordance with these requirements, an electronically steerable compact antenna system capable of beam-switching in the azimuth plane is proposed. The design uses a monopole antenna as the main radiator surrounded by metasurface-based electronically reconfigurable reflector elements designed for the sub-6GHz range. The reflector elements use a reconfigurable capacitively loaded loop (CLL) which can be electronically activated to work as an artificial magnetic conductor (AMC). The design offers a digitally controllable directional radiation pattern covering all 360° in the azimuth plane with a step-size of 30°, a directional gain of ≥4.98 dBi and a high front-to-back lobe ratio (FBR) of ≥14.9 dB. The compact and modular nature of the design combined with the use of commercial off-the-shelf (COTS) components and 3D-printing makes the design low-cost and easier to integrate with various internet of thing (IoT) applications.

## Highlights

• Artificial magnetic conductor (AMC) with the electronic activation mechanism
• Electronically steerable radiation pattern covering the complete horizontal plane
• Digitally controllable steering system using conventional embedded electronics
• Compact, modular and low-cost design using Arduino nano and 3D-printing techniques

## Graphical abstract

**Figure:**
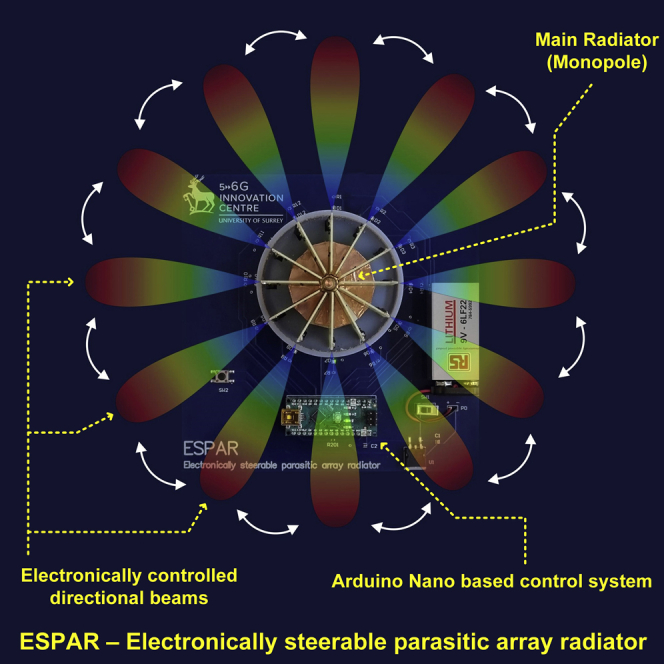

## Introduction

The use of wireless communication networks continues to grow rapidly with more and more users and devices getting added every day. This has created the demand for an improved system capacity and high quality of service (QoS) as we step into the 5G and beyond era. It is being foreseen that smart technologies such as smart antenna systems, transmission surfaces, and reconfigurable intelligent surfaces (RIS) with dynamic beam steering capabilities would be vital elements of such future networks.^1^^,^^2^^,^^3^ Smart antenna systems typically rely on either an adaptive array or beam-switching systems with the former offering better performance than the latter but at the expense of higher complexity and implementation cost. This is majorly due to the use of phase shifters in such adaptive-array antennas which are costly and can suffer from an inherent loss at microwave frequencies.^4^ On the contrary, beam-switching systems such as electronically steerable parasitic array radiators (ESPARs) offer a low-cost and less complex solution to impart beam steering capabilities to an antenna system without the need for expensive phase shifter elements or complex rf chains.^5^

ESPAR systems, originally proposed by Harrington,^6^ typically consist of a single driven element surrounded by several parasitic elements controlled electronically to change the beam direction. Such a feature can be used in typical wireless sensor network (WSN) nodes to transmit (Tx) and receive (Rx) radio frequency (RF) signals from a specific direction quite efficiently as compared to conventional omnidirectional antennas. This is quite useful for internet-of-things (IoT) networks as it can reduce the power consumption of the WSN nodes thereby improving its energy efficiency without compromising on the network performance.^7^ The parasitic elements in combination with the driven element, help to shape the antenna radiation pattern to make it directive toward a particular direction of interest and the fact that it has a single driven element removes the need for bulky phase shifters or complex feed networks.^4^ In conventional ESPAR antennas, this is achieved by controlling the excitation of one or several parasitic elements using electronically tunable reactive loads.^8^ To date, several different realizations of ESPAR have been proposed with different antenna elements. In,^9^ a seven-element ESPAR design is proposed with a single monopole radiator surrounded by six parasitic monopole elements. In,^10^ a thirteen-element ESPAR design has been proposed with one monopole radiator surrounded by twelve parasitic monopole elements providing greater accuracy in the direction of arrival (DoA) estimation. A cavity-backed slot antenna-based ESPAR design is proposed in^11^ providing a wider fractional bandwidth (FBW). Movahedinia et al. have proposed a five-element dielectric resonator antenna (DRA)-based design that is suitable for applications requiring circular polarization.^4^ In,^12^ a liquid antenna-based thirteen-element ESPAR design is presented that uses water cylinders combined with mechanical switches to achieve circular beam-steering capabilities. However, despite such promising designs and features, conventional monopole-based ESPARs suffer from an inherent design constraint due to the 3D nature of their structure. For circular beam-steering characteristics, the parasitic elements need to be kept at ≈0.25λ separation from the main radiator to reduce the coupling effect of the parasitic elements on the main radiator and also to avoid surface currents getting induced in the parasitic elements that could cancel out the currents in the main radiating antenna.^13^ This results in a minimum size constraint on the ESPAR structure which can be considerably high for the sub-6GHz frequency band.

In this article, a twelve-element ESPAR design is proposed in which the surrounding parasitic elements have been replaced with reconfigurable, capacitively loaded loop (CLL)-based reflector elements. CLL structures exhibit properties similar to a perfect magnetic conductor (PMC) and are considered under a class of metamaterials called artificial magnetic conductors (AMCs). Such reflector elements can act as an ideal reflector and can be placed extremely close to the centrally located main radiator providing a directional radiation pattern with a compact size. The design uses digitally controlled PIN diodes to reconfigure the AMC-based reflector elements thereby removing the requirement of varactor-based tunable loads. This makes the proposed design suitable to be interfaced directly to any embedded system-based WSN node as the ON-OFF operation of PIN diodes does not require varying voltage levels. This digital control over the operation of the PIN diodes on all 12 reflector elements facilitates 12 digitally controlled directive radiation patterns uniformly distributed to cover the entire horizontal plane. The proposed ESPAR design further utilizes 3D-printing techniques and commercial off-the-shelf (COTS) components such as Arduino nano to keep the fabrication inexpensive and easy to scale thereby making it an ideal solution for portable, commercial IoT applications where the size and cost is a constraint.

## Theory and design

### Theory of artificial magnetic conductor

**Figure 1:**
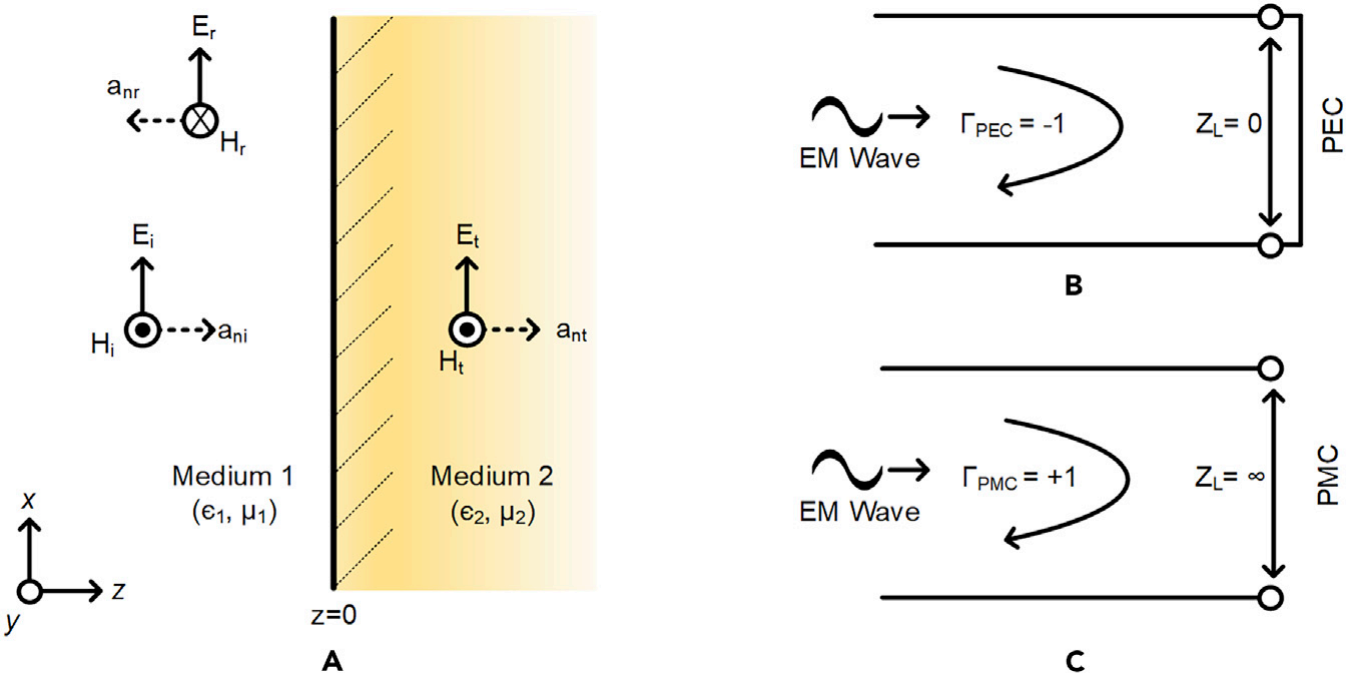
Plane Wave incident normally on a boundary (A) Boundary of two dissimilar media. (B) Case of medium 2 being PEC. (C) Case of medium 2 being PMC.

AMCs are a class of metamaterial^13^ that can produce an in-phase reflection similar to a PMC, which is a fundamental electromagnetic concept, but is usually not observed in naturally occurring materials. Metamaterials have been known to exhibit such beyond nature capabilities and have been used in various applications ranging from microwave to optical frequencies covering a wide range of implementations from metantennas, reflectarrays, radar cross-section reduction, terahertz sensors to optical super lenses.^14^^,^^15^^,^^16^^,^^17^^,^^18^^,^^19^^,^^20^ To further understand how metamaterials can be used to realize AMCs, let us consider an electromagnetic wave traveling through a medium with intrinsic impedance $\left(\sigma_{1}\right)$. When this wave impinges on another medium with a different intrinsic impedance $\left(\sigma_{1}\right)$, it encounters discontinuity resulting in reflections. Let us assume that this electromagnetic wave $\left(E_{i},H_{i}\right)$ traveling through a medium $\left(\epsilon_{1},\mu_{1}\right)$ impinges on another medium $\left(\epsilon_{2},\mu_{2}\right)$ normal to the plane of the medium with the boundary being at Z=0 as shown in Figure 1. The electric and magnetic field intensity phasors for the incident wave can be given by Equations 1 and 2

(Equation 1) $$E_{i}\left(z\right)=a_{x}E_{i0}e^{\left(-j\beta_{1}z\right)}\text{,}$$

(Equation 2) $$H_{i}\left(z\right)=a_{y}\frac{E_{i0}}{\eta_{1}}e^{\left(-j\beta_{1}z\right)}\text{.}$$

where $\eta_{1}$ is the intrinsic impedance and $\beta_{1}$ is the phase constant of medium 1. The value of Z is <0 in medium 1 and >0 in medium 2. At Z=0 lies the discontinuity in the medium which will cause the incident wave to partly reflect in medium 1 and partly transmit into medium 2. The electric and magnetic field intensity phasors for the reflected wave ($E_{r}$, $H_{r}$) can be expressed by Equations 3 and 4

(Equation 3) $$E_{r}\left(z\right)=a_{x}E_{r0}e^{\left(j\beta_{1}z\right)}\text{,}$$

(Equation 4) $$H_{r}\left(z\right)=-a_{y}\frac{E_{r0}}{\eta_{1}}e^{\left(j\beta_{1}z\right)}\text{.}$$

and for the transmitted wave ($E_{t}$, $H_{t}$) by Equations 5 and 6

(Equation 5) $$E_{t}\left(z\right)=a_{x}E_{t0}e^{\left(-j\beta_{2}z\right)}\text{,}$$

(Equation 6) $$H_{t}\left(z\right)=a_{y}\frac{E_{t0}}{\eta_{2}}e^{\left(-j\beta_{2}z\right)}\text{.}$$

It should be noted that the directions of arrows depicting $E_{r}$ and $E_{t}$ have been arbitrarily drawn as they could change directions depending on the constitutive parameters $\left(\varepsilon_{1},\mu_{1},\varepsilon_{2},\mu_{2}\right)$ of the respective two media. At the interface of these two media (Z=0), the tangential components of the electric and magnetic field intensities should be continuous, therefore by applying boundary conditions the magnitudes of $E_{r0}$ and $E_{t0}$ can be determined by using Equations 7 and 8.

(Equation 7) $$E_{r0}=\frac{\eta_{2}-\eta_{1}}{\eta_{2}+\eta_{1}}E_{i0}\text{,}$$

(Equation 8) $$E_{t0}=\frac{2\eta_{2}}{\eta_{2}+\eta_{1}}E_{i0}\text{.}$$

These lead us to the ratios $E_{r0}/E_{i0}$ and $E_{t0}/E_{i0}$ which are also called the reflection coefficient (Γ) and transmission coefficient (τ) respectively and can be expressed in terms of the intrinsic impedances by Equations 9 and 10

(Equation 9) $$S_{11}=\Gamma =\frac{E_{r0}}{E_{i0}}=\frac{\eta_{2}-\eta_{1}}{\eta_{2}+\eta_{1}}\text{,}$$

(Equation 10) $$S_{21}=\tau =\frac{E_{t0}}{E_{i0}}=\frac{2\eta_{2}}{\eta_{2}+\eta_{1}}\text{.}$$

It should be noted that the reflection coefficient (Γ) can be either a positive or a negative quantity depending on the properties of the two media and can even have a complex value depicting variation in both magnitudes as well as phase of the reflected electromagnetic wave as compared to the incident wave. This has been explained in Section case of Medium 2 being perfect electric conductor (PEC) and Section case of Medium 2 being perfect magnetic conductor (PMC) by considering two common theoretical boundary conditions.

#### Case of medium 2 being perfect electric conductor

PEC is one of the most commonly used boundary conditions in electromagnetics in which the electric field component tangential to the surface is zero i.e., $\hat{n}\times E=0$ and $\hat{n}\cdot H=0$^21^ where $\hat{n}$ is the unit vector pointing normal to the boundary. It is quite often used to model a lossless metallic surface (with infinite electrical conductivity) in microwave and antenna applications to simplify numerical computations. PEC imposes symmetry for magnetic fields and anti symmetry for electric fields thereby inducing currents within, which always balances out the currents flowing into the PEC. Now assuming medium 1 $\left(\sigma_{1}\right)$ to be air and medium 2 $\left(\sigma_{2}\right)$ to be a perfect electric conductor (i.e. $\sigma_{2}\to \infty$), the intrinsic impedance of medium 2 can be approximated to $\eta_{2}\to 0$ thus resulting in Equation 9 becoming as follows:

(Equation 11) $$S_{11}=\Gamma =\frac{0-\eta_{1}}{0+\eta_{1}}\Rightarrow \Gamma =-1\text{,}$$

Equation 11 shows that in case of a PEC, the reflected wave bouncing off the PEC surface will be reflected with a phase shift of 180° signified by the negative sign of the reflection coefficient (Γ).

#### Case of medium 2 being perfect magnetic conductor

PMC is another boundary condition used in electromagnetics in which the tangential component of the magnetic field is zero i.e., $\hat{n}\times H=0$ and $\hat{n}\cdot E=0$^21^ where $\hat{n}$ is the unit vector pointing normal to the boundary. This implies that the surface current density is zero and that no electric currents (volume, surface, or edge) can flow inside a PMC boundary as that would violate current conservation. For this reason, PMCs can also be interpreted as high impedance surfaces, which in addition to setting the surface current density to zero also impose symmetry for electric fields. Now assuming medium 1 $\left(\sigma_{1}\right)$ to be air and medium 2 $\left(\sigma_{2}\right)$ to be a perfect magnetic conductor. Since surface currents cannot flow inside a PMC, the intrinsic impedance of medium 2 can be approximated as $\eta_{2}\to \infty$. Thus considering $\eta_{2}\gg \eta_{1}$, Equation 9 becomes as follows:

(Equation 12) $$S_{11}=\Gamma =\frac{\eta_{2}}{\eta_{2}}\Rightarrow \Gamma =+1\text{,}$$

Equation 12 shows that in case of a PMC, the impinging electromagnetic wave will be reflected but with a 0° phase shift signified by the positive sign of the reflection coefficient (Γ). It should be noted that PMCs are only considered as a special boundary condition as there are no naturally occurring materials yet known that can exhibit such properties but if discovered, PMCs could give way to compact antennas with reduced mutual coupling and improved front-to-back ratio (FBR).

### Practical realizations of artificial magnetic conductor

PMC materials do not exist in nature as there are no free moving magnetic charges, however, an alternative approach of designing an engineered metasurface^22^ that could offer characteristics of a PMC material using the concept of high surface impedance can be modeled by considering an array of parallel resonant LC circuits. We know from circuit theory that such LC circuits shunt the current through the inductor at low frequencies and through the capacitor at high frequencies but at a certain resonance frequency $\left(\omega_{0}\right)$ they offer a very high impedance governed by the respective L and C values which can be calculated by $\omega_{0}=1/\sqrt{LC}$.^13^ Such circuits can be implemented on a substrate for microwave frequencies by designing a specific two-dimensional geometry that would behave as a parallel resonant LC circuit in a specific bandgap thereby offering high impedance to the surface currents formed as a result of the impinging electromagnetic waves.^23^ Such artificially engineered surfaces which behave similar to a PMC and offer an in-phase reflection are known as artificial magnetic conductors (AMCs). Contrary to the conventional PEC reflectors, AMC-based reflectors can be positioned extremely close to the antenna. This is primarily due to the fact that surface currents induced in AMCs due to the impinging electromagnetic waves are more in-phase than out of phase and hence placing them extremely close to the radiating element does not short it out resulting in a compact antenna design.^13^ This is the main concept that has been used in this work for designing the AMC-based reflector elements to direct the antenna radiation in a particular direction.

**Figure 2:**
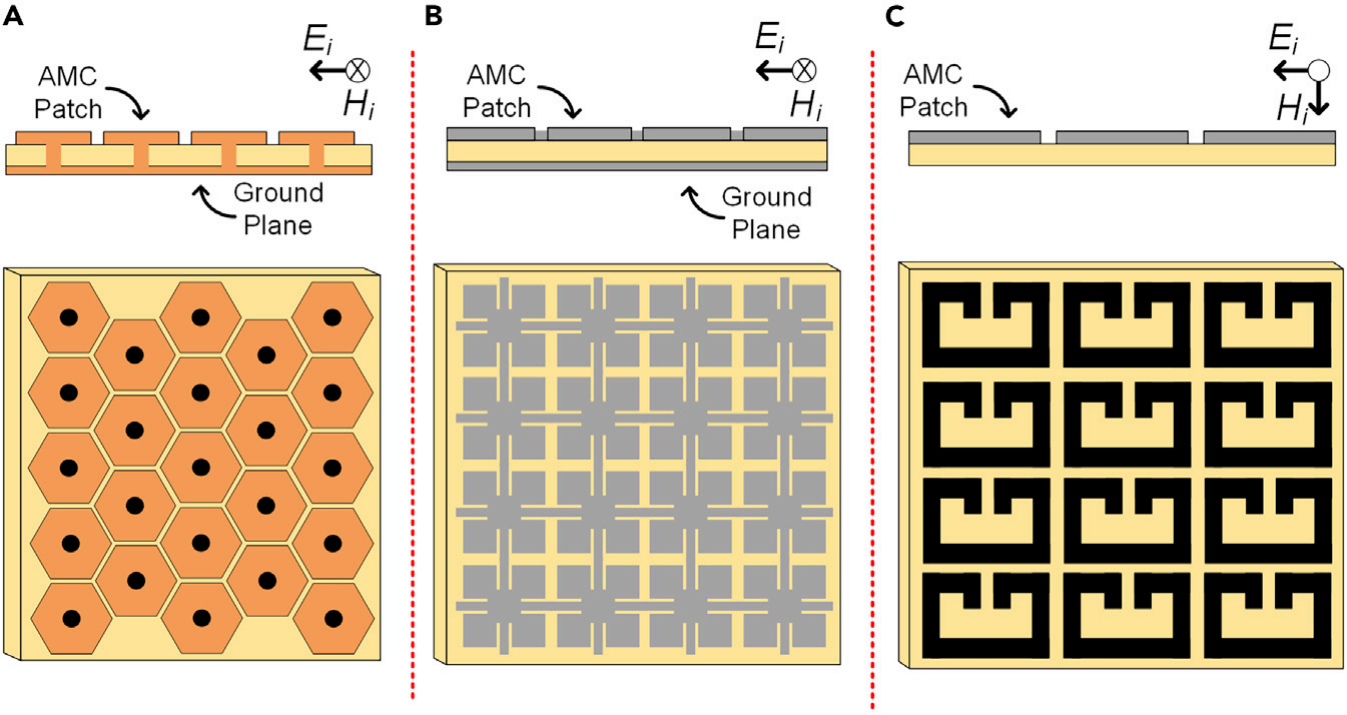
Popular AMC realizations (A) Mushroom realization. (B) UC-PBG realization. (C) CLL-based volumetric realization.

Although various such structures have been proposed in the existing literature but the most popular and widely used are the mushroom realizations,^13^ the uniplanar compact photonic bandgap (UC-PBG) realizations^24^ and the capacitively loaded loop (CLL) structures as shown in Figure 2.^25^ The mushroom realization consists of a plane of periodically located elements backed by a ground plane with a dielectric separating the two planes connected by vias as shown in Figure 2A. The UC-PBG-based realizations, do not require any vias and instead achieve AMC behavior by utilizing periodic elements with complex shapes as shown in Figure 2B. Though such structures act as an AMC but having a finite PEC-based ground introduces some challenges such as image and scattering-induced currents at the back side of such structures increasing the probability of surface waves and lowered front-to-back ratio (FBR). Additionally, having such a continuous PEC-based ground plane makes the design bulky and if placed extremely close, can still cause shorting out of the radiating element. However, in the CLL-based volumetric realization, such complications are not present as it achieves AMC behavior using a simplified version of the split-ring resonators (SRRs) called the capacitively loaded loops (CLLs) as shown in Figure 2C. This type of AMC structures do not require a common ground plane and hence is considered suitable for ESPAR applications.

### The reconfigurable artificial magnetic conductor reflector element

**Figure 3:**
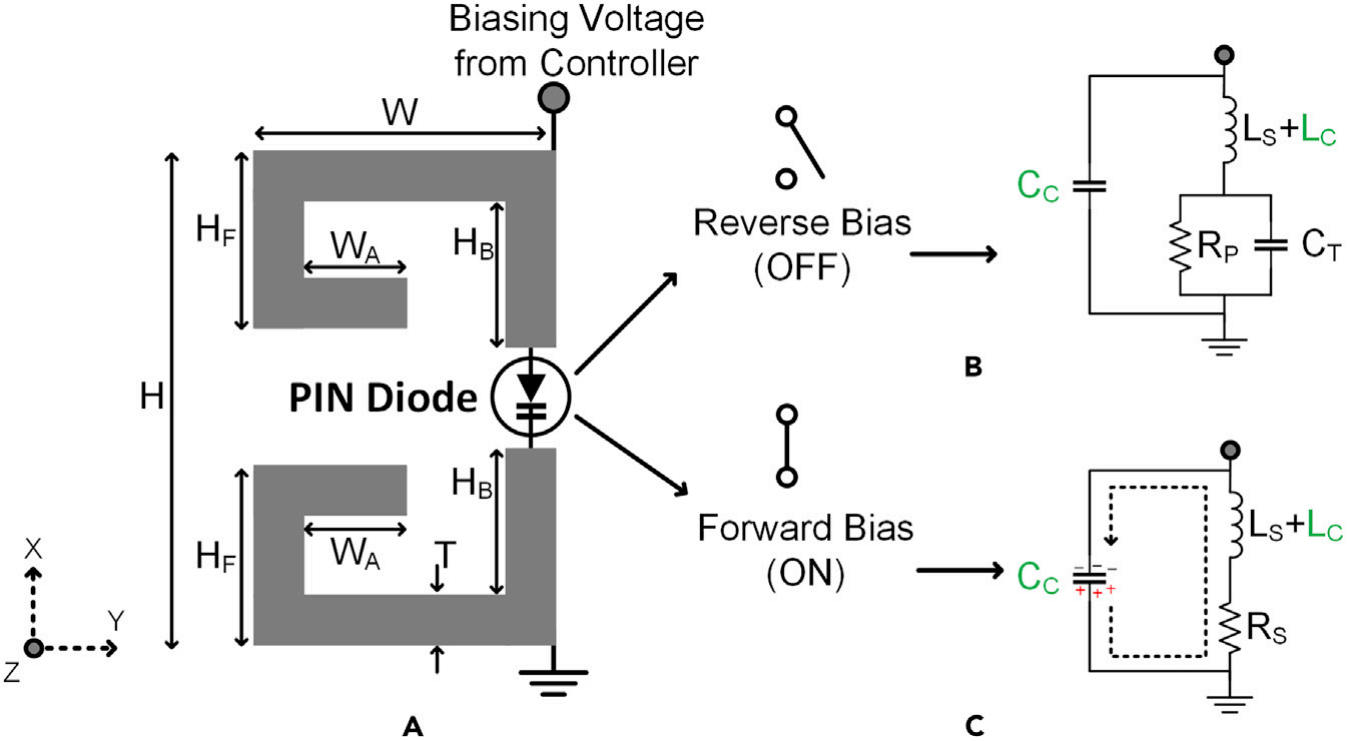
The proposed reconfigurable AMC unit cell (A) Electronically activated CLL structure. (B and C) (B) Equivalent circuit diagram of CLL with PIN diodes reverse biased (C) Equivalent circuit diagram of CLL with PIN diodes forward biased.

**Figure 4:**
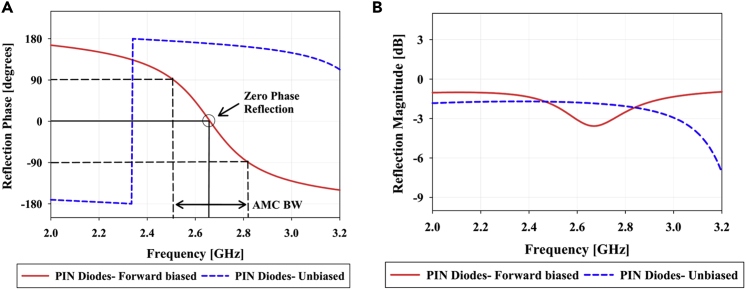
Reflection characteristics $\left(S_{11}\right)$ of the proposed AMC unit cell (A) Reflection phase $\left(∠S_{11}\right)$ vs frequency. (B) Reflection magnitude $\left(\left|S_{11}\right|\right)$ vs frequency.

The AMC realizations discussed in Section practical realizations of AMC cannot be used directly for ESPAR operation as they lack reconfigurability which is vital for switching the radiation pattern of the ESPAR antenna in a specific plane. For this purpose, a modified CLL-based AMC unit cell is proposed as shown in Figure 3A. The CLL loop has been split into two parts connected using the PIN Diode BA89202VH6127XTSA1 of package TSLP-2-1 which offers extremely low forward resistance ($R_{S}$ <1Ω) and high reverse parallel resistance ($R_{P}$ >100kΩ). By controlling the biasing voltage applied across the PIN diode, it can be operated as an ON and OFF switch. This controlling of the diode enables the structure to function as an electronically controlled CLL loop thereby offering AMC properties of zero phase crossing when the PIN diode is in the forward-biased state and acting as a non-CLL structure when the diode is in an unbiased state.This means the reflector element can be activated or deactivated by controlling the biasing of the PIN diodes and hence can be used to dynamically direct the direction of radiation of an antenna. The proposed reconfigurable AMC unit cell element along with its equivalent circuit diagram is shown in Figure 3 where $L_{C}$ & $C_{C}$ represent the equivalent inductance and capacitance respectively of the CLL structure and $R_{P}=100$ kΩ, $R_{S}=0.36\Omega$, $L_{S}=0.4$ nH and $C_{T}=1$ pF represent the typical values of reverse parallel resistance, series forward resistance, lead inductance and reverse capacitance of the PIN diode respectively. The operating mechanism of this reconfigurable unit cell is based on the fact that the series forward resistance of the PIN diode is extremely low as compared to the reverse parallel resistance ($R_{S}\ll R_{P}$). Thus, when a positive voltage is applied to the anode of the PIN diode to forward bias it, the diode behaves as a near short circuit thereby completing the CLL loop and activating its AMC behavior. On the contrary, when this positive voltage is removed, the PIN diode no longer works as a short circuit thereby breaking the CLL loop and deactivating it as an AMC reflector. It must be noted that for practical applications, the zero-phase crossing point might not be the most optimal point of operation for any AMC reflector design due to various practical constraints. Hence, the design and placement of the AMC reflector need to be slightly tuned in accordance with the specific application for which it is being designed.^25^^,^^26^^,^^27^ The final design parameters of the proposed unit cell are H=9.64 mm, $H_{F}=4.12$ mm, $H_{B}=3.68$ mm, W=6.02 mm, $W_{A}=1.87$ mm, T=1.08 mm which have been tuned to work at 2.66 GHz for an ESPAR application. The AMC characteristics of a unit cell can be verified by analyzing its reflection coefficient for a uniform incident plane wave. The amplitude and phase response of the proposed reconfigurable AMC unit cell are given in Figure 4 for different biasing states of the PIN diode. The phase response shows a zero-phase crossing point at 2.66 GHz when the PIN diode is forward biased resulting in AMC behavior at that frequency.

**Figure 5:**
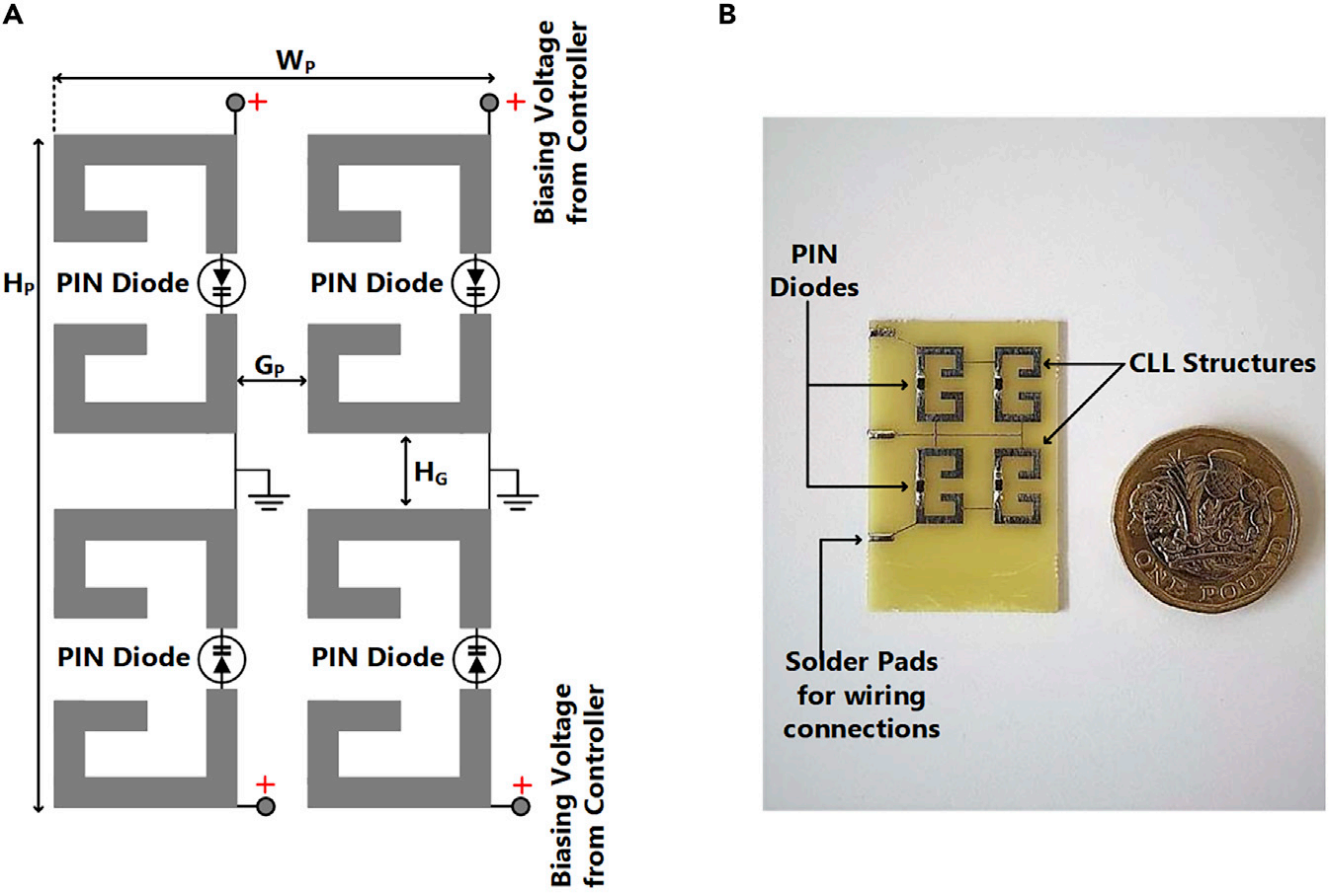
The complete reconfigurable AMC-based reflector element for the ESPAR (A and B) (A) The reflector element with a 2×2 configuration (B) The fabricated reflector element with bias lines for electronically controlled activation.

The AMC unit cell was arranged in a 2×2 configuration for the reflector element as shown in Figure 5 which was fabricated using an FR-4 substrate of thickness of 0.8 mm and dielectric permittivity $\left(\varepsilon_{r}\right)$ of 4.3. All the CLL structures are printed on one side of the substrate and a bias line of 0.2 mm thickness was added to allow biasing voltage to be applied across the PIN diodes. The final design parameters of the reflector element consisting of 4 CLL structures are $H_{P}=22.6$ mm, $W_{P}=15.95$ mm, $G_{P}=3.91$ mm, $H_{G}=3.32$ mm. The PIN diodes have been oriented in such a way that a minimalist circuit layout is used. The top and bottom bias lines have been connected to the source of biasing voltage and the middle bias line acts as a common dc ground for the PIN diodes.

## Fabrication and measurements

### The electronically steerable parasitic array radiators design and implementation

**Figure 6:**
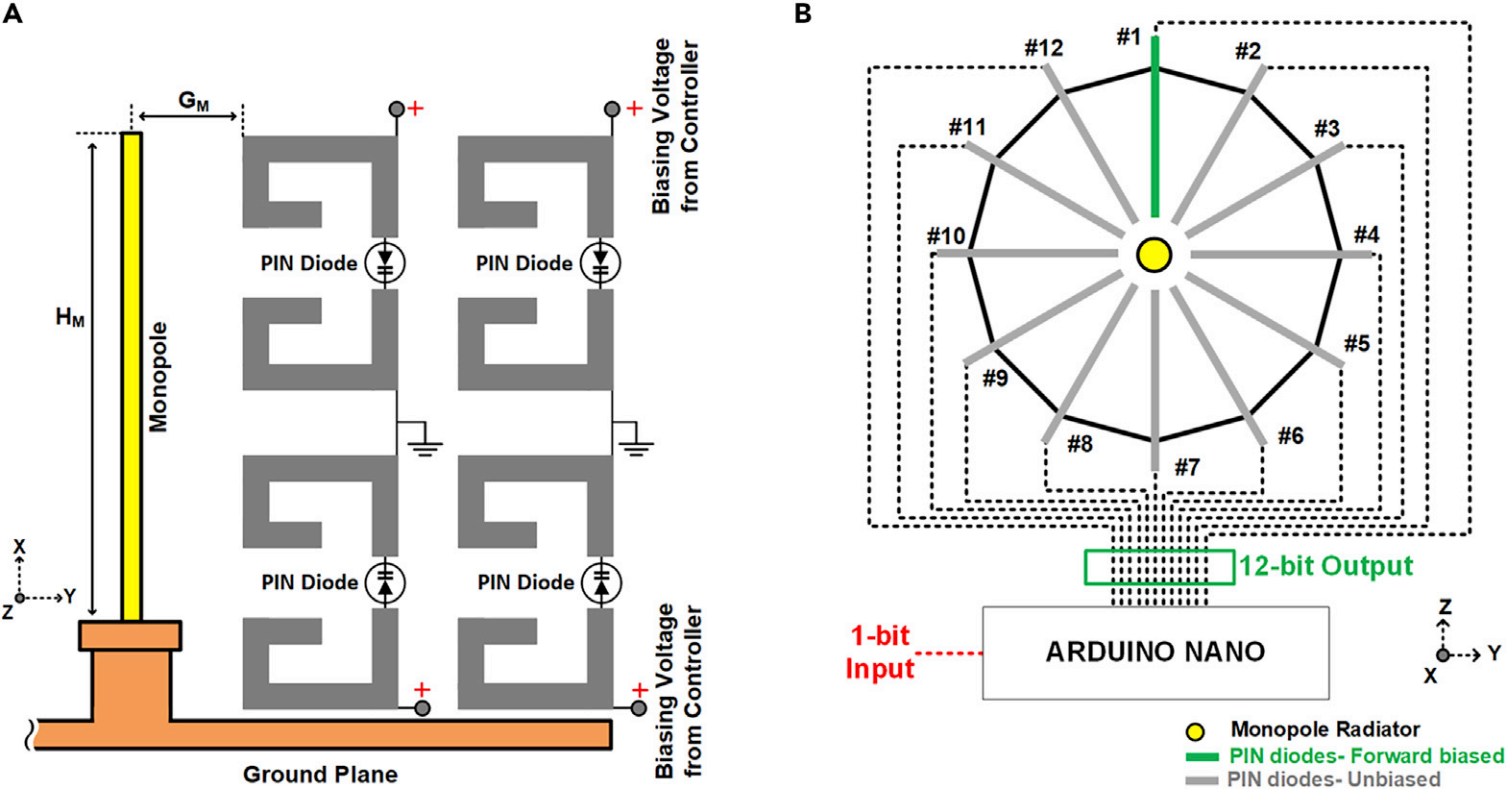
Arrangement of the proposed ESPAR antenna (A and B) (A) The reconfigurable reflector element placed adjacent to the central monopole (B) Top view showing the arrangement of all twelve reconfigurable reflector elements being controlled by a 12-bit steering vector (figure shows $V_{\max}^{1}=\left[100000000000\right]$) generated by an Aurdino Nano-based control system.

**Figure 7:**
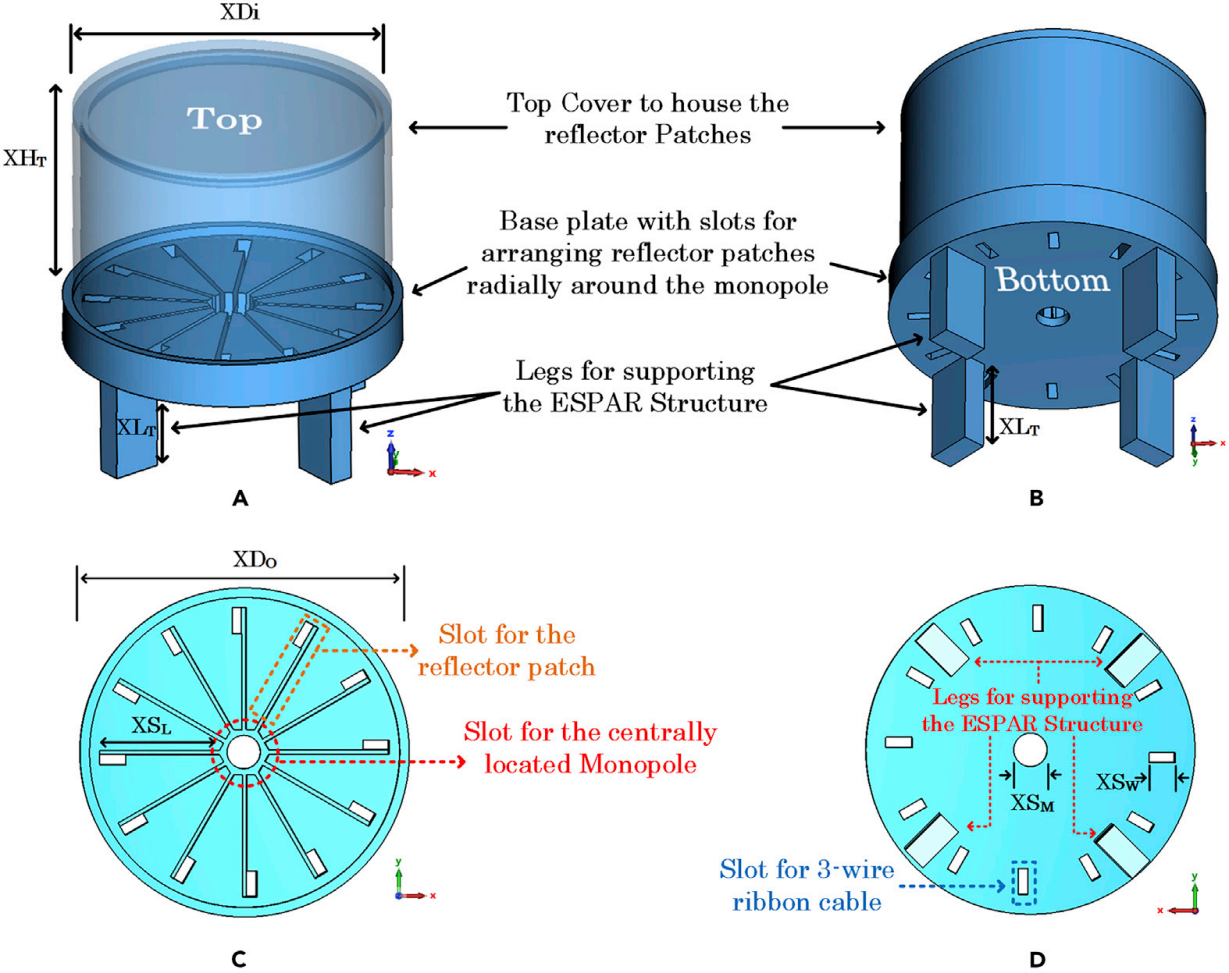
The 3D-printed housing to arrange the reflector elements in the radial configuration around the monopole antenna (A) Perspective top view of the full 3D-printed structure. (B) Perspective bottom view of the full 3D-printed structure. (C) Top view of the base plate with slots for the reflector elements. (D) Bottom view of the base plate with slots for the 3-wire ribbon cables.

**Figure 8:**
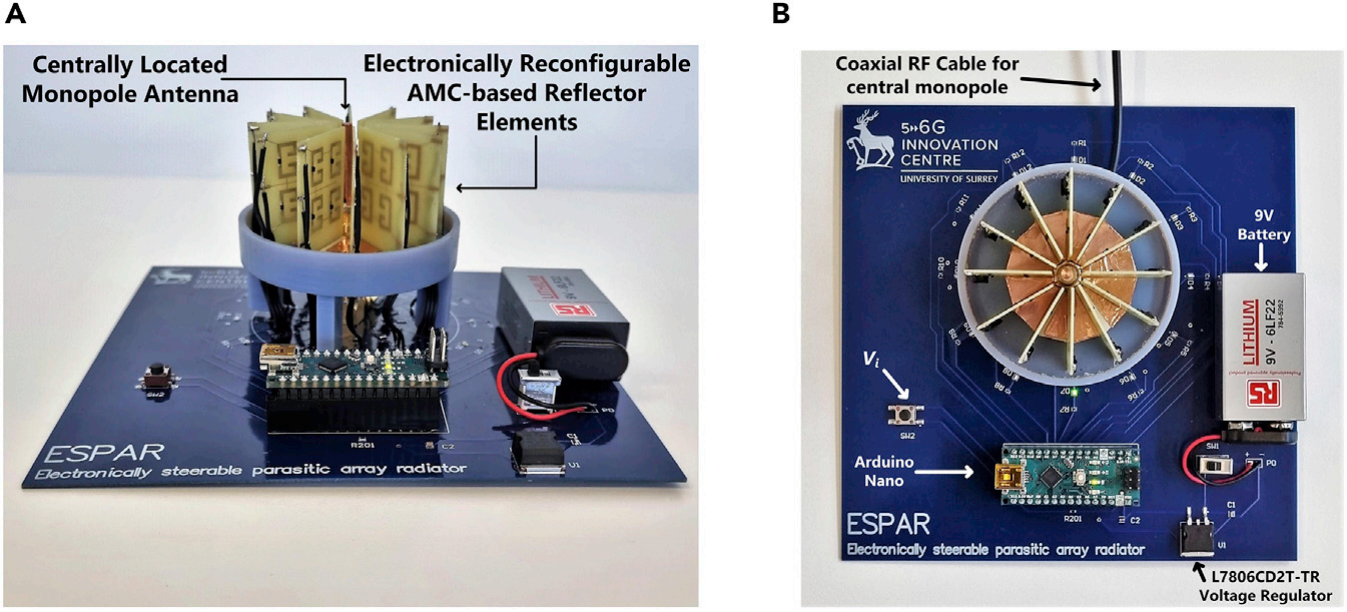
The fabricated prototype of the complete ESPAR system (A) Side view. (B) Top view.

A typical ESPAR antenna consists of a centrally located radiating element surrounded by parasitic elements to reconfigure the radiation pattern in a specific plane. The proposed ESPAR design consists of a quarter-wave monopole fed by a centrally located coaxial connector and surrounded by N=12 reconfigurable reflector elements. Each reflector element has been realized with a 0.8 mm thick FR-4 substrate with all of the elements being radially arranged around the monopole of height $H_{M}=23.7$ mm at a distance of $G_{M}=6.57$ mm from the central axis of the monopole as shown in Figure 6. As metasurface-based designs are highly sensitive to the positioning and alignment therefore, arranging the 12 reflector elements in a radial configuration around the monopole required a custom-built design that could hold the structure firmly. For this reason, an encasing has been designed and 3D-printed to fix the the complete ESPAR assembly around the central monopole. The material used was “VeroBlue RGD84” which was printed using the 3D-printing facility at the faculty of engineering and physical sciences (FEPS) at the university of surrey, U.K. As shown in Figure 7, the 3D-printed housing can be divided into two main sections: (i) the top section consisting of a cylinder of height $XH_{T}=37.9$ mm with a top lid of diameter $XD_{i}=59$ mm, acting as a cover to protect the reflector elements. (ii) The bottom base plate of diameter $XD_{O}=62.2$ mm with 12 slots of length $XS_{L}=24.5$ mm in which the AMC-based reflector elements can be inserted. The whole structure is supported by 4 legs of height $XL_{T}=20$ mm keeping the ESPAR antenna elevated above the Arduino-based beam switching system. At the center of the base plate is a hole of diameter $XS_{M}=6.7$ mm to insert the centrally radiating monopole. The 3D-printed structure offers a robust design to arrange the reconfigurable reflector elements extremely close to the centrally located monopole. Each element has been connected to the Arduino-based beam switching controller using a 3-wire ribbon cable coming out of the slot of size $XS_{W}=5.3$ mm on the 3D-printed base plate facilitating the individual control of the reflector elements. The Arduino Nano has been programmed to take 1-bit input $\left(V_{i}\right)$ and give a 12-bit output that can be sequentially changed. This 12-bit output corresponds to a steering vector $V_{\max}^{n}=\left[v_{1}\;v_{2}\ldots .v_{n}\ldots v_{12}\right]$, where $v_{n}$ denotes the state of the $n^{th}$ reflector with “1” representing AMC mode and “0” representing non-AMC mode. The state depicted in Figure 6B, where the reflecting element #1 is in AMC mode and the remaining reflecting elements are in non-AMC mode, can be represented by an equivalent steering vector of $V_{\max}^{1}=\left[100000000000\right]$. Each distinct value of the steering vector corresponds to a distinct directional radiation pattern with the main beam pointing at an angle denoted by $\theta_{\max}^{n}$. The state of $V_{i}$ can be changed by using a switch which is connected to ground on one side and to the $V_{i}$ pin of the Arduino Nano on the other side. Each time the switch is pressed, the state of $V_{i}$ toggles from “1” to “0” triggering a change in the steering vector $V_{\max}^{n}$ thus making it possible to cycle through all the possible steering vector values. An important factor to note about the proposed ESPAR is the highly modular and adaptive nature of the design. In case a change in the operating frequency is required, unlike most of the other designs present in the existing literature which might require a complete redesign, the proposed ESPAR would only require a change of monopole and the reflector elements. The new reflector elements could be slotted into the existing 3D-printed structure and can be connected to the existing Arduino-based steering system without requiring any other change. Another modularity is related to the digital control of the system due to the use of PIN diodes. Conventional ESPAR designs rely on base-loaded varactor diodes with voltage-controlled tunable loads for steering the beam direction. This approach is not suitable for typical IoT applications due to its higher power requirements and the varying voltage levels required for controlling the varactor diodes.^8^ Since the proposed ESPAR system only requires the activation of a single reflector element for each directional radiation pattern using PIN diodes, the power requirements are much less and suitable for IoT applications. Lastly, since the design is Arduino-based, which supports serial communication, the prototype ESPAR system can take serial input in addition to the designed 1-bit input methodology from any embedded system having a serial interface. This enables reconfiguring the ESPAR to any possible value of the steering vector $V_{\max}^{n}$ randomly without the need for sequential shifting. All these factors make the design versatile, highly modular, and suitable for typical IoT applications. The completely manufactured prototype of the proposed ESPAR design along with the custom-built Arduino-based switching system is shown in Figure 8.

### Results and discussion

**Figure 9:**
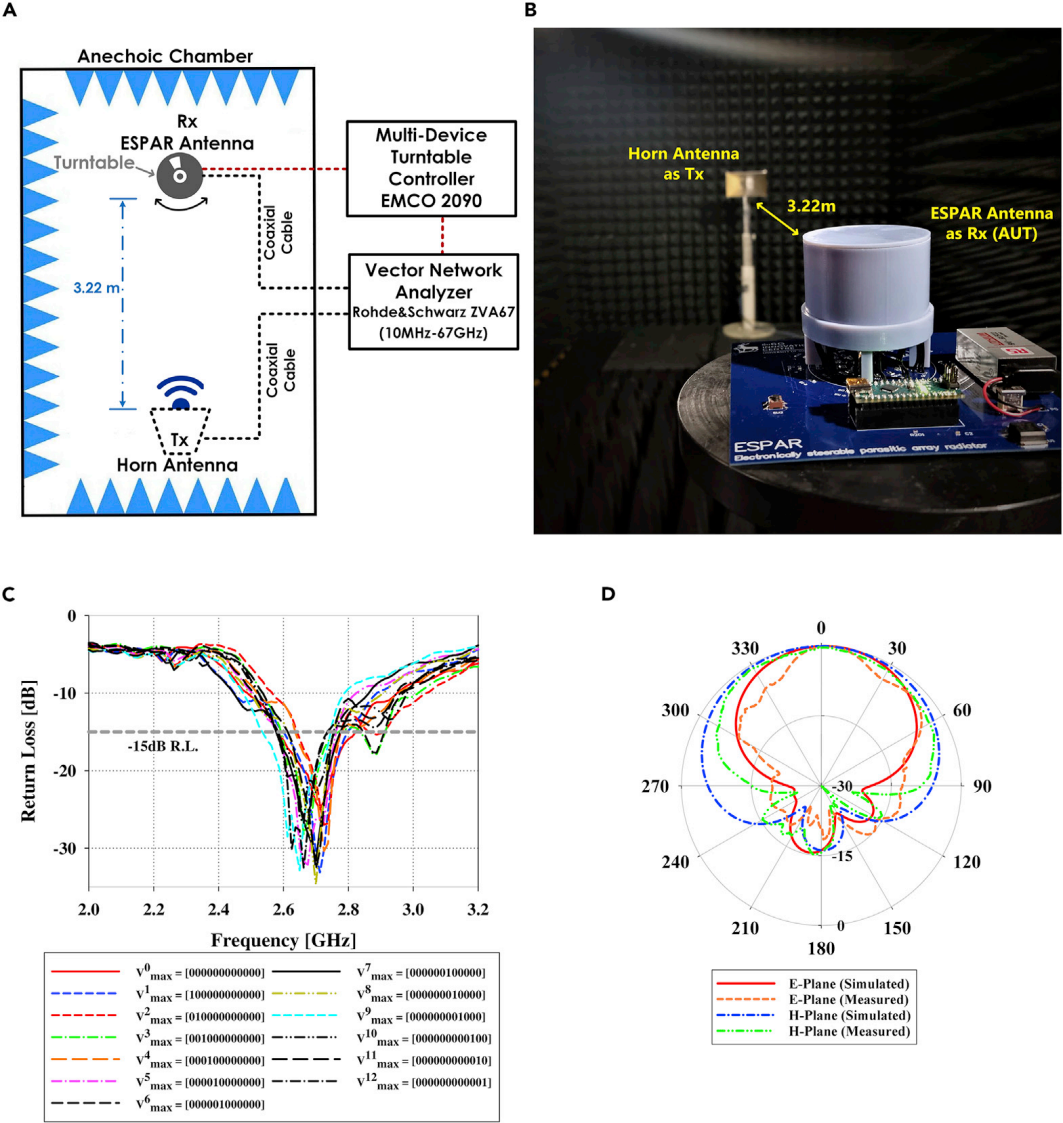
Measurement setup and results (A and B) Measurement setup and results of the fabricated ESPAR prototype (A) Setup to measure the beam switching characteristics (B) View of the ESPAR antenna inside the anechoic chamber. (C) Return loss measured for all values of steering vector $\left(V_{\max}^{n}\right)$. (D) Radiation Pattern for steering vector $V_{\max}^{7}=\left[000000100000\right]$ in elevation plane (E-Plane) and azimuth plane (H-Plane).

A prototype of the proposed ESPAR design was fabricated and tested in an anechoic chamber as shown in Figure 9B. The measurement setup is presented in Figure 9A and consists of a Rohde & Schwarz-ZVA67 vector network analyzer (VNA) transmitting a signal at 2.65 GHz using a standard horn antenna and then receiving the signal using the antenna under test (AUT) which is the proposed ESPAR antenna. The ESPAR antenna is positioned on a turntable controlled by the multi-device turntable-controller EMCO-2090 and can be rotated in complete 360° direction in the horizontal plane with a step size of 1°. The reflection coefficient $\left(\left|S_{11}\right|\right)$ of the centrally located monopole antenna was measured for various values of the steering vector $V_{\max}^{n}$ and the results are presented in Figure 9C. The impedance matching of the monopole antenna is observed to deviate slightly for different values of the steering vector $V_{\max}^{n}$. This is due to the deviation in the alignment of the different reflector elements with respect to the monopole antenna owing to the finite accuracy of the 3D-printing used to make the antenna housing. This creates minor changes in the placement of the AMC-based reflectors thereby causing such variations. However, the antenna still offers an overlapped 15 dB return loss bandwidth of 100 MHz from 2.65 GHz to 2.75 GHz and twelve distinct radiation patterns for each value of the steering vector.

**Figure 10:**
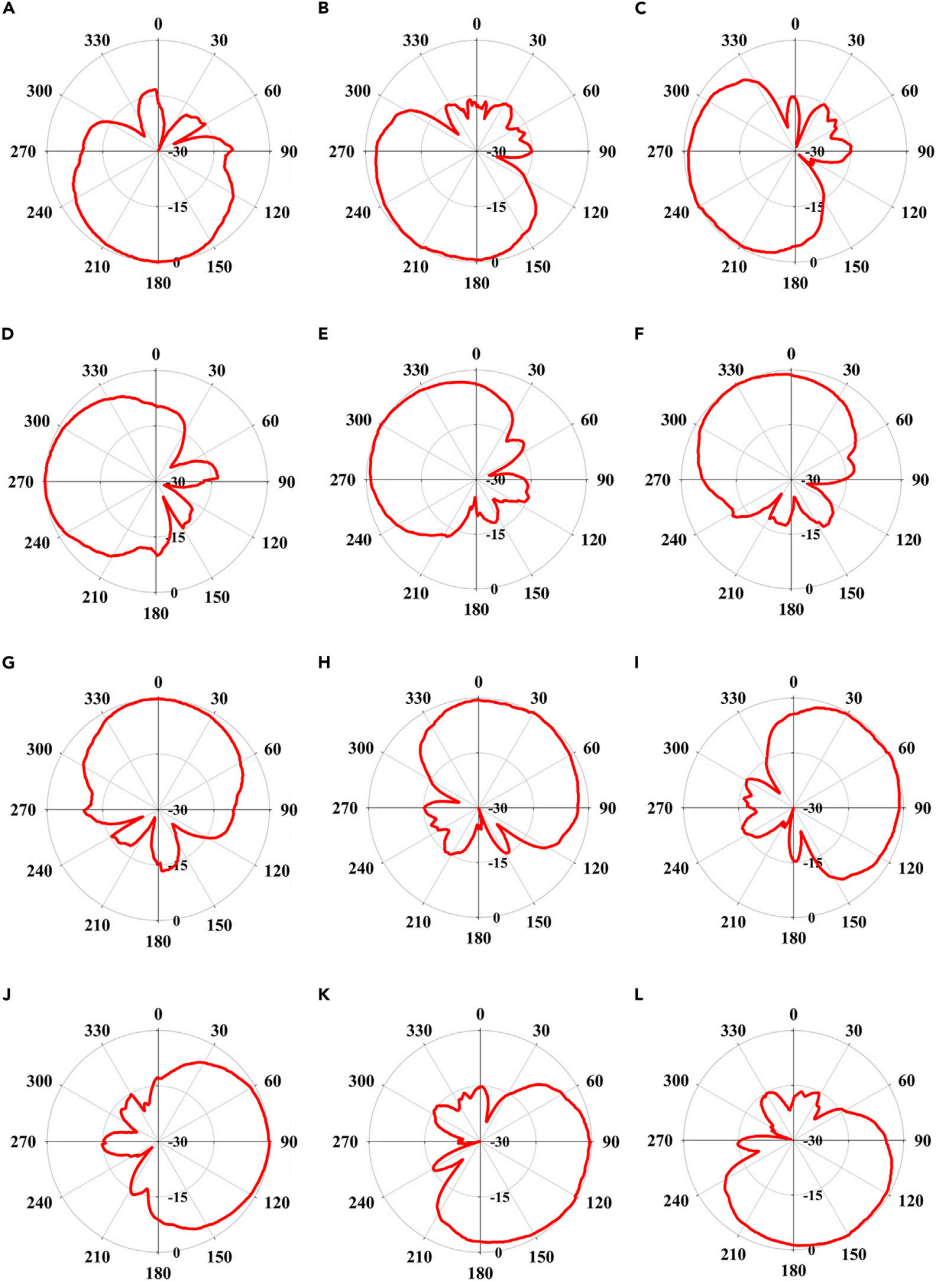
Measured ESPAR radiation patterns in azimuth plane for different values of steering vector $V_{\max}^{n}$ (A) $V_{\max}^{1}$= [100,000,000,000]. (B) $V_{\max}^{2}$= [010,000,000,000]. (C) $V_{\max}^{3}$= [001,000,000,000]. (D) $V_{\max}^{4}$= [000,100,000,000]. (E) $V_{\max}^{5}$= [000,010,000,000]. (F) $V_{\max}^{6}$= [000,001,000,000]. (G) $V_{\max}^{7}$= [000,000,100,000]. (H) $V_{\max}^{8}$= [000,000,010,000]. (I) $V_{\max}^{9}$= [000,000,001,000]. (J) $V_{\max}^{10}$= [000,000,000,100]. (K) $V_{\max}^{11}$= [000,000,000,010]. (L) $V_{\max}^{12}$= [000,000,000,001].

The direction of the radiation pattern can be switched in the azimuth plane over the complete 360° range with twelve different directional radiation patterns. This reconfiguration of the radiation pattern is controlled by the Arduino-based custom-designed beam switching system which can generate various different values of the steering vector $V_{\max}^{n}$. The E-plane and H-plane far-field radiation patterns corresponding to the steering vector $V_{\max}^{7}=\left[000000100000\right]$ are presented in Figure 9D which uses N=7 reflector element in AMC mode. The proposed ESPAR antenna offers a highly directional far-field radiation pattern with a main beam directional gain of 4.98-5.66 dBi which ensures that the IoT node can direct it’s radiation toward the direction of interest thereby reducing the transmit power requirements. The design exhibits a high FBR of 14.9-17.6 dB which ensures a considerable attenuation of the unwanted signals coming from the backward direction thereby reducing the probability of packet collisions and lowering the overall power requirements of the IoT node even further.^8^ The design also offers a half-power beamwidth (HPBW) of 84°–118° in the azimuth plane which ensures that the ESPAR can do a DoA estimation with minimum error. The far-field radiation pattern in the azimuth plane for various different steering vector values is presented in Figure 10 and summarized in Table 1. The impact of the finite fabrication accuracy can also be seen in the far-field radiation characteristics. Any shift in the alignment of the reflector elements with respect to the monopole can potentially cause a shift in the phase of the electromagnetic wave reflecting from the AMC-based reflector. This ultimately results in slight deviations among the far-field radiation characteristics for different values of the steering vector ($V_{n}^{\max}$). However, the deviation is nominal if the phase shift does not exceed the AMC bandwidth which is ±π/2 from the zero phase crossing as image currents are more in-phase within this band than out-of-phase.^13^

**Table 1:** Summary of the far-field radiation characteristics of the proposed ESPAR antenna in the azimuth plane for different values of steering vector $\left(V_{\max}^{n}\right)$

Element in AMC mode (#n)	Steering vector ($V_{\max}^{n}$)	Main beam direction ($\theta_{\max}^{n}$)	Main beam gain ($G_{\max}^{n}$)	Front-to-back ratio (FBR)	H-Plane beamwidth (HPBW)
1	[100,000,000,000]	181°	5.66 dBi	17.1 dB	86°
2	[010,000,000,000]	211°	5.03 dBi	15.1 dB	118°
3	[001,000,000,000]	242°	5.09 dBi	17.6 dB	117°
4	[000,100,000,000]	267°	5.38 dBi	17.3 dB	85°
5	[000,010,000,000]	299°	5.11 dBi	17.5 dB	92°
6	[000,001,000,000]	330°	5.28 dBi	17.1 dB	89°
7	[000,000,100,000]	2°	5.35 dBi	15.1 dB	84°
8	[000,000,010,000]	32°	4.98 dBi	14.9 dB	118°
9	[000,000,001,000]	63°	5.02 dBi	14.9 dB	117°
10	[000,000,000,100]	93°	5.21 dBi	15.2 dB	98°
11	[000,000,000,010]	124°	4.99 dBi	14.9 dB	117°
12	[000,000,000,001]	151°	5.05 dBi	15.2 dB	107°

To illustrate the advantages of the proposed ESPAR design, a comparison has been done with some of the other reported ESPAR designs that offer similar 360° beam steering capabilities in at least one plane as given in Table 2. The design reported in^28^ offers the highest number of directional radiation patterns with a high directional gain but is considerably bigger than the proposed design and requires 2-5 steering elements to be in active state for every radiation pattern. This can cause a higher power consumption to operate such as the design that requires multiple elements to be in the active state which might not be ideal for low power IoT applications. The design reported in^12^ is a unique design that offers 12 directional radiation patterns and comparable gain but is based on a liquid antenna design that is considerably bulky, fragile, and would require frequent maintenance making it unsuitable for compact IoT applications. The design reported in^29^ is low-profile but has a considerably bigger footprint in the horizontal plane as compared to the proposed design. Although it offers a higher gain but offers only 8 directive radiation patterns as compared to 12 in the proposed design and also requires 3 active elements for each directive radiation pattern leading to a higher power consumption. The design concept reported in^8^ offers a total of 12 directive radiation patterns with a good directional gain and FBR but requires 4-5 steering elements to be in active state for every radiation pattern as compared to only 1 active steering element required in the proposed design. Finally, the designs reported in^30^^,^^31^ have comparable compact size but offer only 4 and 6 directional radiation patterns respectively that too with a lesser directional gain as compared to the proposed ESPAR design. Thus, from this comparison, it is clear that the proposed ESPAR design offers a compact size with lower power requirements for its operation without compromising much on the ESPAR antenna performance.

**Table 2:** Comparison of the proposed design with other ESPAR designs reported in the existing literature

Reference	Horizontal span	Parasitic elements	Steering method	Active steering elements	Directional radiation patterns	Main beam gain	HPBW in steering direction	FBR in steering direction
28	1.06λ	Parasitic Monopoles	SPDT Switches	2–5	6×3	7.7–8.1 dBi	88°–100°	8–20 dB^a^
12	0.86λ	Water filled Monopole Reflectors	Water Valves	5	12	4.2–5.8 dBi	–	13–15 dB^a^
29	1.31λ	Loaded-disk Microstrip	PIN Diodes	3	8	7.3–7.5 dBi	66.2°–69.1°	12–15 dB a
8	0.59λ	Horizontal Slots	PIN Diodes	4–5	6×2	3.4–4.9 dBi	108°–160°	14.8–31.8 dB
30	0.42λ	Parasitic Rings	PIN Diodes	1	4	3.5 dBi	120°	20 dB
31	0.4λ	Folded Monopoles	Varactor Diodes	3	6	3.3–4 dBi	–	20 dB
This work	0.39λ	Reconfigurable AMC-based Reflectors	PIN Diodes	1	12	4.9–5.6 dBi	84°–118°	14.9–17.6 dB

a Value estimated from measured radiation patterns reported in the literature.

### Conclusion

A compact ESPAR antenna that can perform beam-switching in the horizontal plane using metasurface-based electronically reconfigurable AMC reflector elements with twelve distinct main beam directions is proposed. To the best of the author’s knowledge, this is the first such implementation of an ESPAR antenna where reconfigurable AMC-based reflector elements have been used to steer the beam in the horizontal plane. The proposed ESPAR design offers a 15 dB return loss bandwidth of 100 MHz ranging from 2.65 GHz to 2.75 GHz. The design offers twelve directional radiation patterns with a main beam directional gain of ≥4.98 dBi and an FBR of ≥14.9 dB that can be digitally controlled by an Arduino-based or any similar embedded system design capable of handling a 12-bit steering vector. The proposed design achieves this by digitally controlling the functioning of the twelve CLL-based reflector elements by forward biasing or unbiasing the onboard PIN diodes thereby activating or deactivating the AMC-based reflector elements respectively. Since the number of reflector elements biased for any value of steering vector is just 1, therefore the power requirements of the proposed design are extremely small making it suitable for low-power WSN nodes and the use of COTS combined with 3D-printing techniques makes this design extremely versatile, modular and suitable for various IoT applications.

### Limitations of the study

The proposed metasurface-based ESPAR design can only use those AMC structures that follow a volumetric design approach without a common ground. The two main reasons behind this are: (i) parasitic elements need to be arranged in a radial pattern to achieve a 360° beam switching and that (ii) AMC-based reflectors need to be placed very close to the centrally located radiating antenna due to an in-phase reflection. Also, due to the highly resonant nature of the AMC structures, the design is very sensitive to fabrication tolerances and requires a high level of fabrication accuracy to reproduce consistent results.

## STAR★Methods

### Key resources table

**Table:** 

REAGENT or RESOURCE	SOURCE	IDENTIFIER
**Software and algorithms**		

CST Studio Suite	Dassault SystÃlmes	CST 2021
MATLAB	The MathWorks, Inc	R2021a

**Other**		

VeroBlue	StrataSys	RGD840
Vector Network Analyzer	Rohde&Schwarz	ZVA67
Multi-Device Positioning Controller	ETS-Lindgren EMCO	2090

### Resource availability

#### Lead contact

Further information and resources related to this study will be fulfilled by the lead contact Vikrant Singh (vikrant.singh@surrey.ac.uk) upon reasonable request.

#### Materials availability

This paper did not generate new unique reagents.

### Method details

#### Numerical analysis

All the numerical analysis required for this work was conducted by CST Design Studio Suite 2021. Firstly, a modified version of the CLL-based AMC unit cell was designed that could exhibit in-phase reflection at 2.66 GHz. The CLL loop was then split up into two-halves connected by a PIN Diode that offered extremely low forward resistance and high reverse parallel resistance. The CLL loop could be completed by biasing the PIN diode thereby activating in-phase reflection and therefore making it digitally controllable. The reflection phase and amplitude of the AMC unit cell was tuned so that it could work as an ideal reflector when activated. Secondly, twelve reconfigurable reflector elements were designed using this AMC unit cell arranged in a 2×2 configuration. All these twelve elements were then placed radially around the monopole and were activated in a sequential manner to study their impact on the resulting antenna performance and radiation characteristics.

#### Sample fabrication

A quarter-wave monopole was placed at the center of the ESPAR structure fed by a centrally located coaxial cable. The monopole was surrounded by twelve reconfigurable reflector elements connected to the aurdino based beam-switching system. The reflector elements were designed to be digitally controlled using a 12-bit steering vector governed by the aurdino based system. The complete antenna unit comprising of the monopole and the twelve reconfigurable reflector elements was arranged together using a 3D-printed cylindrical structure shown in Figure 7. The reflector elements were slotted inside the 3D-printed structure and were connected to the electronic beam switching system using 3-wire ribbon cables to enable the digital control of their activation and deactivation as AMC reflectors.

#### Experimental measurements

Under normal circumstances a monopole antenna exhibits an omnidirectional radiation pattern in the azimuth plane but by activation and deactivating the reflector elements in AMC mode, a directional radiation pattern can be generated directed toward a specific angle governed by the position of the active reflector element. This was experimentally verified by placing the fabricated prototype on a turntable which was controlled by a multi-device positioning controller. The 12-bit steering vector $\left(V_{\max}^{n}\right)$ was then changed in such a way that the 12 AMC reflector elements could be activated one-by-one in a sequential manner. The turntable was rotated a complete 360° in the azimuth plane for each value of the steering vector $\left(V_{\max}^{n}\right)$ to measure the corresponding radiation pattern of the ESPAR antenna.

## Data Availability

• Data reported in this paper will be shared by the lead contact upon request. • This paper does not report original code. • Any additional information required to reanalyze the data reported in this paper is available from the lead contact upon request.
